# Combined Mini-Parasep SF and Nanogold Immunoassay Show Potential in Stool Antigen Immunodetection for Giardiasis Diagnosis

**DOI:** 10.1038/s41598-019-55492-1

**Published:** 2020-03-30

**Authors:** Ahlam F. Mohram, Waleed E. Elawamy, Marwa M. Nageeb, Hemat S. Ali, Shereen M. Kishik

**Affiliations:** 1 Department of Parasitology, Banha University Faculty of Medicine, Banha, Egypt; 2 Pathology Department (Microbiology Unit), Jouf University College of Medicine, Sakaka, Saudi Arabia

**Keywords:** Biological techniques, Microbiology

## Abstract

Covalent loading or directional binding of biomolecules on gold nanoparticles (AuNPs) could lead to better results than simple direct adsorption for an enhanced ELISA application. The use of Mini-Parasep solvent-free (SF) without ether or ethyl acetate for the clean and efficient concentration of protozoa cysts, it is a single-use device for *in vitro* diagnostic use only. In this work, we used Mini-Parasep SF for the detection of *giardia* cysts in comparison to direct smear and Merthiolate-Iodine Formaldehyde Concentration (MIFC) technique in addition to its use in antigen detection by AuNPs biomolecule loading using rabbit polyclonal antibodies (pAb) against purified *Giardia* antigen (PGA). As a result, Mini-Parasep SF was the most effective method for *Giardia* cyst detection and regarding optimization of Mini-Parasep antigen detection, our data showed increased sensitivity and specificity of nano-sandwich ELISA to 92% and 94% respectively and increased positive predictive value (PPV) and negative predictive value (NPV) to 88.64% and 95.91% respectively. In conclusion, this research provides that Mini-Parasep SF concentrator enhanced *Giardia* cyst detection and improved antigen preparation for AuNPs sandwich ELISA in giardiasis diagnosis. The advantages of this method are the short assay time and the raised accuracy of antigen detection providing concentrated samples without the risk of solvent use and being a disposable Mini-Parasep it helps in *giardia* antigen purification as well as raising the sensitivity and specificity of ELISA through binding AuNPs.

## Introduction

Direct microscopic examination of stool specimens is still representing the gold standard in the diagnosis of giardiasis although it is time-consuming and requires skillful technicians. Multiplex PCR or antigen-based detection tests show current methods of interest replacing the microscopy in Giardia diagnosis being technically less complex and requiring limited training^1^. To reduce diagnostic delays, antigen-based detection diagnostic tests take a shorter time to give results within 15–20 minutes demonstrating that stool antigen immunodetection-based modalities are rapid, reliable, sensitive, specific and standardized tests for routine diagnosis of *Giardia*^2^.

It was found that comparing antigen-based detection tests to wet mount microscopy and concentration steps saved time and was suitable for laboratories having little microscopic skills in the scope of diagnosis and not able to afford the high costive and sophisticated molecular diagnostic methods^1^.

Mini-Parasep SF fecal concentrator provides a single collection vial to allow the collection and concentration of stool samples, it was designed to replace the formalin-ether concentration technique and distributed routinely in laboratories. Due to the high efficacy in detecting parasites, formalin-ether concentration technique is commonly used in diagnostic purposes and epidemiological studies but it has formalin and ether which carry significant health hazards for laboratory workers and environmental harms being fiscally detrimental so Mini-Parasep SF was introduced as a laboratory diagnostic kit in spite of lack of proving its efficacy or sensitivity and are still many questions to ask about its use instead of conventional concentration methods^3^.

Mini-Parasep solvent-free technique when compared to the current standard stool concentration methodology was evaluated having less debris, sedimentation clarity and background uniformity of material for microscopic detection of parasites while on comparison of Midi-Parasep to Mini-Parasep solvent-free fecal parasite concentrators methods, it was found that samples with small numbers of parasites can be diagnosed as positive by using Midi-Parasep concentrator with ethyl acetate that can be missed by Midi-Parasep solvent-free system^4^.

In recent years, there is an increased rate of using stool antigen immunodetection-based assays for the detection of *Giardia* infections in humans, widely in diagnostic laboratories in both underdeveloped and developing countries and this is because of their higher sensitivity compared to conventional microscopic diagnostic methods. Antigen detection modalities have rapid diagnostic capabilities and comparable sensitivity and specificity to those of stool-based PCR methods for the diagnosis of giardiasis^5^.

ELISA technique is considered as the most important diagnostic technique in biosciences nowadays, being based on the specific recognition potential of antigenic molecular design as an epitope in facing a specific antibody^6^. Plasmic gold was utilized as energy harvester as well as energy converter helping in the separation of electron-hole pairs due to effect of generated H_2_O_2_ of enzyme sandwiched immunoreaction on exposure to the radiation of a 980 nm laser and this H_2_O_2_ was generated by the help of AuNPs heavily functionalized with polyclonal antibodies in presence of glucose oxidase, thereby resulting in the enhanced photocurrent via capturing holes to promote the separation of electron-hole pairs^7^.

With this methodology, many diseases, alimentary troubles, and small molecules can be detected and diagnosed. For this reason, improving the detection level, sensitivity or reducing the test time will be an important goal in several fields, so nanotechnology is considered a target field for developing the diagnostic techniques being based on functionalization of nanoparticles from gold to carbon as antibody carriers^6^. Enzyme free immunoassays bring a promising and innovative thinking for the detection of low-abundance biomarkers through a near-infrared activated non-enzymatic signal off photoelectrochemical immunoassay for ultrasensitive detection of serum proteins by upconversion nanoparticle structures coupled with copper ions^8^.

The aim of this work is to isolate GA by the Mini-Parasep concentrator technique and using it in coproantigen-based immunodetection of giardiasis with the application of traditional sandwich ELISA in comparison to nano-magnetic beads sandwich ELISA immunoassay thus improving the diagnostic modalities of antigen-based immunodetection assays in giardiasis.

## Materials and Methods

### Ethical approval

Informed consent was obtained from all individual participants included in the study. This study was approved by the Banha Ethical Committee at Banha University and Theodore Bilharz Institute Ethical Committee. In our study, the guidelines as per the Declaration of Helsinki for involving human participants were followed. The ethical committee of Theodore Bilharz Research Institute approved the use of rabbit in this study according to the international guiding principles for biomedical research involving animals as issued by the international organizations of medical sciences.

### Study subjects

Fecal specimens in this study were collected from human subjects with diarrheic stool (n = 81), subjects without diarrheic stool but have parasitic infections with signs of parasitism (n = 20) and subjects who are normal controls (n = 20). *Giardia* infected human subjects were males (n = 41) and female (n = 26) on microscopic examination using Mini-Parasep. Human subjects stool specimens were collected from Theodore Bilharz Institute, Giza, Egypt and Banha University Hospital, Banha, Egypt. No funding was given to us for this work.

### Stool specimens and processing

Fecal samples (2–5 grams) were collected aseptically from the subjects, transported immediately to the laboratory in Theodore Bilharz Institute, Giza, Egypt, kept in cold place until examined.

### Stool microscopy and Mini-Parasep kit

Specimens were initially surveyed by direct smear method according to Cheesbrough^9^ and MIFC method according to previously reported studies^10,11^ then the samples were examined by Mini-Parasep SF fecal parasite concentrator (manufactured by *DiaSys Europe Ltd, Berkshire, Wokingham, United Kingdom, product code 148800*) which is single use tube. According to the manufacturer’s instructions, the lid was unscrewed and 3.3 ml of 10% buffered formalin + one drop of Triton X were added to the mixing tube. A level scoop of the fecal sample was introduced by using the spoon on the end of the Mini-Parasep filter. The sample was mixed thoroughly with the Parasep spoon. Mini-Parasep was immediately sealed by screwing in the filter thimble and conical tube. The mixture was vortexed with sedimentation cone pointing upwards and Mini-Parasep was then inverted to allow the mixture tube filtered through the filter thimble. Mini-Parasep was then centrifuged at 500 g for 2 min, it fits all 15 ml centrifuge buckets. The mixing tube and filter thimble were unscrewed and discarded. All the liquid above the sediment was poured off and 1 ml water was added to sediment. The sediment was re-suspended with water by shaking. The sediment then was pipetted to a slide for microscopic examination^12,13^.

### Preparation and purification of GA

*G. lamblia* cysts were collected from fresh, refrigerated stool specimens, previously verified to be *G. lamblia*-positive by microscopy. Approximately 10 grams of stool were suspended in 5 ml of distilled water, mixed well by stirring, filtered through two layers of cheesecloth, and centrifuged at 500 to 700 × g for 5 min. The pellet was discarded, and the supernatant was subjected to 50% ammonium sulfate precipitation followed by centrifugation at 10,000 × g for 20 min. Supernatants were suspended in 10 mM phosphate buffer (pH 6.8) containing 0.1% Triton X-100 and were sonicated on ice with a minimum of eight 10-s pulses from a Branson cell disrupter. Complete disturbance of the cyst preparation was detected by light microscopy. Sonicated preparations were centrifuged at 12,000 × g for 5 min, the pellet was suspended in distilled water, extensively dialyzed at 4 °C against distilled water containing 0.1% ammonium hydroxide. The dried material was suspended in 5 ml of 50 mM Tris buffer (pH 8.2) containing 1 mM phenyl-methyl-sulfonyl fluoride and 5 mM EDTA and stored at −20 °C until needed for affinity chromatography^14^.

### Preparation of pAb against PGA

Just before immunization, rabbits' sera were assayed by ELISA for *Giardia* antibodies and cross-reactivity with other parasites. Rabbits were injected intramuscularly, with 1 mg of PGA mixed by 1:1 *Freund’s* complete adjuvant. Then, two booster doses were given at one-week intervals after the primary injection, each was 0.5 mg antigen emulsified in equal vol. of *Freund’s* incomplete adjuvant^15^. One week after the last booster dose, the rabbits’ sera were obtained and pAb fraction was purified by 50% ammonium sulfate precipitation method^16^. More purification of pAb was performed by 7% caprilic acid method^17^. The IgG was produced with high degree of purification, but a minimal amount of serum protein may contaminate. Partially purified pAb was further adsorbed with fetal calf serum (*FCS; Seromed*) to eliminate any non-specific binding with bovine antigen^18^.

### Preparation of AuNPs

**Figure 1:**
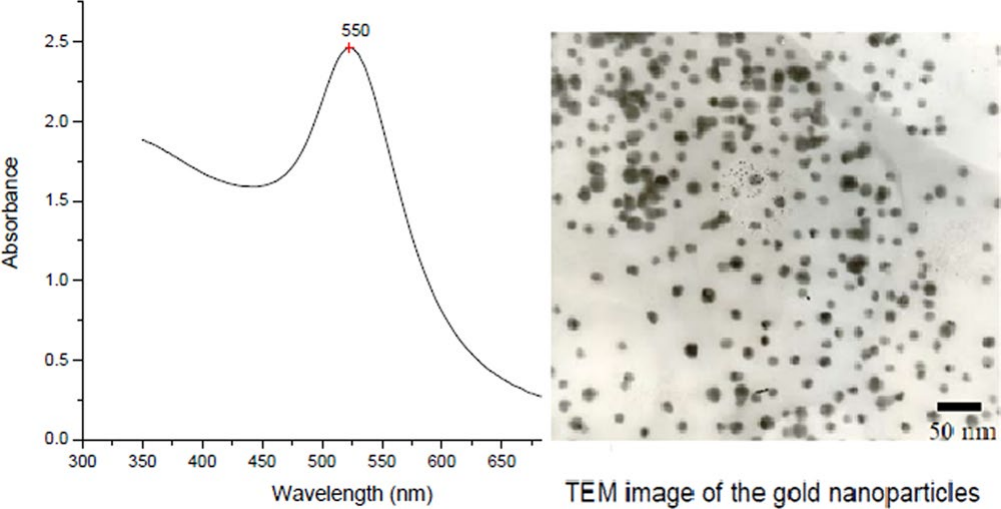
Characterization of AuNPs by UV-Vis spectrophotometer and TEM.

AuNPs were functionalized according to Omidfar *et al*.^19^. After the synthesis of the AuNPs, the next step was their characterization (Fig. 1) according to Rajeshkumar *et al*.^20^.

### Loading of AuNPs to pAb

Conjugation of AuNPs to pAb was done by adding 5 mg of pAb in 2 ml of phosphate buffer (pH 7.5) to 5 ml pH-adjusted AuNPs solution. The mixture was gently mixed for 3 h, and then the residual surface of the AuNPs was blocked by adding 1 ml of 10% Bovine Serum Albumin (BSA) solution to the mixture. It was then incubated for 20 min at room temperature before three times centrifugation at 13,000 rpm for 45 min at 4 °C. After the last centrifugation, the pellets were re-suspended in 2 ml phosphate buffer (pH 7.2, 0.01 M) containing 1% BSA and 0.05% sodium azide. Conjugated AuNPs-pAb solution was stored at 4 °C before being used^21^.

The conjugation of antibodies to the surface of AuNPs was reported by three interactions: a) the binding between amino acid sulphur atoms of antibodies and the gold conducting electrons; b) attraction between hydrophobic parts of antibody and metal surface of AuNPs; c) ionic attraction between the positively charged antibodies and the negatively charge gold. Adapter molecules like Streptavidin and Biotin, chemisorption vial thiol derivatives, and bifunctional linkers are the ways used in chemical interaction between antibodies and nanoparticles surface thus helping conjugation by covalent and non-covalent immobilization modes^22^.

### Optimization of Working Dilutions of Coating AuNPs-pAb and Determination of the Lower Detection Limit by Indirect ELISA

Chequer-board titration was used as an indicator for optimum dilutions of the conjugated AuNPs-pAb solution being tested in different concentrations against known positive and negative stool samples. A standard curve was set up using serial dilutions of PGA from 3 ug/ml to 0.6 ng/ml. The lower detection limit of PGA concentrations was determined by Optical density (OD) readings equal to 492 nm^23,24^.

### Detection of Giardia antigen (GA) in stool samples of giardiasis patients by sandwich ELISA using AuNPs-pAb

Antigen detection was done by sandwich ELISA according to *Blagg et al*.^18^, and modified by Kamel *et al*.^25^. AuNPs conjugated pAb were used to coat microtitration plates (*Thomas Scientific, Swedesboro, New Jersey, United States*) in the form of 100 ul/well in 0.1 M carbonate buffer pH 9.6 and left overnight at room temperature. 2.5% FCS was used to block plates and added by 200 µl/well in 0.02 M phosphate-buffered saline with 0.05% Tween 20 (PBS/T) pH 7.2 for 2 h at 37 °C.

100 µl of stool supernatant samples diluted 1:4 in diluent buffer were pipetted into the wells of the blocked plate in duplicate and incubated for 2 h at 37 °C. Plates were washed 3 times with PBS/T. 100 µl of 1/1000 horseradish peroxidase-conjugated pAb in diluent buffer was added to each well and the plate was incubated for 1 h at 37 °C. The wells were then washed 5 times, 3 min each. The reaction was visualized by the addition of 100 µl/well of O-phenylene diamine (*Sigma Co., St Louis, MO, USA*) for 20 minutes in the dark at room temperature. The reaction was stopped by adding 50 µl/well of 8 NH_2_S0_4_ and plates were read at 492 nm using an ELISA reader (*Microplate Reader, Bio-Rad, in Richmond, California, USA*).

### Statistical analysis

Results of nano-sandwich-ELISA were evaluated compared to the results of sandwich ELISA. All data were submitted to Microsoft Excel 2013 and analyzed using SPSS (version 17 windows). The cut off value was calculated as the mean OD readings of positive and negative samples ± standard deviations of the mean (± SD). Sensitivity (%) = T_1_/(T_1_ + F_2_) × 100, specificity (%) = T_2_/(T_2_ + F_1_) × 100, positive predictive value (%) = T_1_/(T_1_ + F_1_) × 100 and negative predictive value (%) = T_2_/(T_2_ + F_2_) × 100, where T_1_ = True positive, T_2_ = True negative, F_1_ = False positive and F_2_ = False negative^26^.

## Results

### Comparison of 3 diagnostic methods to detect Giardia lamblia (*G. lamblia*)

**Figure 2:**
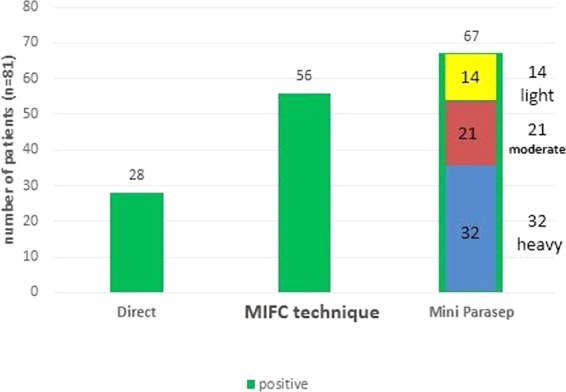
Detection of *G. lamblia* cysts in stool samples of human by direct smear, MIFC technique and Mini-Parasep.

Based on microscopic examination using direct smear 28 samples (34.6%) were positive from 81 suspected to have *G*. *lamblia* cysts or trophozoites while using MIFC technique 56 samples (61.9 %) were positive from 81 suspected samples, while based on Mini-Parasep 67 samples (82.7%) were positive from 81 suspected samples. According to Mini-Parasep, the stool samples of infected humans with *G. lamblia* cyst were classified into heavy, moderate and light infections samples, 32 of samples were heavy, 21 were moderate and 14 were a light infection (Fig. 2).

### Age and sex distribution of patients infected with giardiasis

Male cases had the highest percentage of infection with *G. lamblia* in age group 0–10 years (37.5%) followed by age groups 21–30 years (20%) on microscopic examination using Mini-Parasep (Table 1).

**Table 1:** Age and sex distribution of giardiasis infected patients on microscopic examination using Mini-Parasep.

Age group in years	Male		Female		Total number	Total Percent
	No	%	No	%		
0–10	15	22.5	10	14.9	25	**37.4**
10–20	4	5.9	1	1.5	5	**7.4**
21–30	11	16.5	6	8.9	17	**25.4**
31–40	4	5.9	4	5.9	8	**11.8**
41–50	3	4.6	3	4.5	6	**9.1**
51–60	4	5.9	1	1.5	5	**7.4**
Above 60	0	0	1	1.5	1	**1.5**
Total	41	61.3%	26	38.7%	67	100%

### Reactivity and specificity of PGA by sandwich ELISA

**Figure 3:**
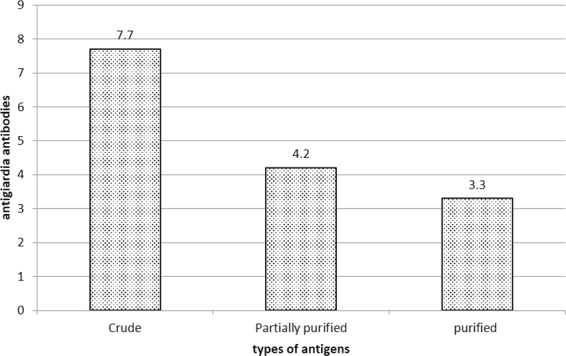
Detection of GA by sandwich ELISA.

The antigenicity and specificity of PGA were characterized by sandwich ELISA. Stool samples from *G. lamblia* infected patients gave less positive results against PGA than those against crude antigens and partially purified antigens (Fig. 3).

### PGA Detection by both sandwich and nano-sandwich ELISA in human stool samples

In sandwich ELISA technique of our work, positive cases were detected by cut off value at 0.27 so in group (I) we found 68 positive cases out of 81 diarrheic patients while the other parasitic group showed 4 cases only out of 20 at the borderline of cut off value. Giardia lamblia infection by the same technique appeared below the cut off value in all the 20 negative control patients. While in the nano-sandwich ELISA technique of this work, in group (I) diarrheic patients we found 74 positive cases of *G. lamblia* infection out of 81 samples, and one out of 20 of other parasitic group was at the borderline of the cut off value. All the 20 negative controls were below the cut off value (Table 2).

**Table 2:** Results of sandwich ELISA and nano-sandwich ELISA in diagnosis of *G. lamblia*.

Variable groups	Sandwich ELISA				Nano-sandwich ELISA			
	Positive cases		Negative cases		Positive cases		Negative cases	
	No	Mean ± SD	No	Mean ± SD	No	Mean ± SD	No	Mean ± SD
Group I: (diarrheic patients n = 81)	**68**	1.09 ± 0.25	**13**	0.19 ± 0.16	74	1.31 ± 0.29	**7**	0.24 ± 0.15
Group II: (other parasitic infections n = 20)	**3**	0.27 ± 0	**9**	0.21 ± 0.05	1	—	**11**	0.15 ± 0.05
Entamoeba histolytica n = 12								
Enterobius vermicularis n = 8	**1**	—	**7**	0.11 ± 0.06	0	—	**8**	0.09 ± 0.04
Group III: (Healthy controls n = 20)	**0**	—	**20**	0.17 ± 0.06	0	—	**20**	0.21 ± 0.02

### Sensitivity, specificity, positive predictive value (PPV) and negative predictive value (NPV)

**Figure 4:**
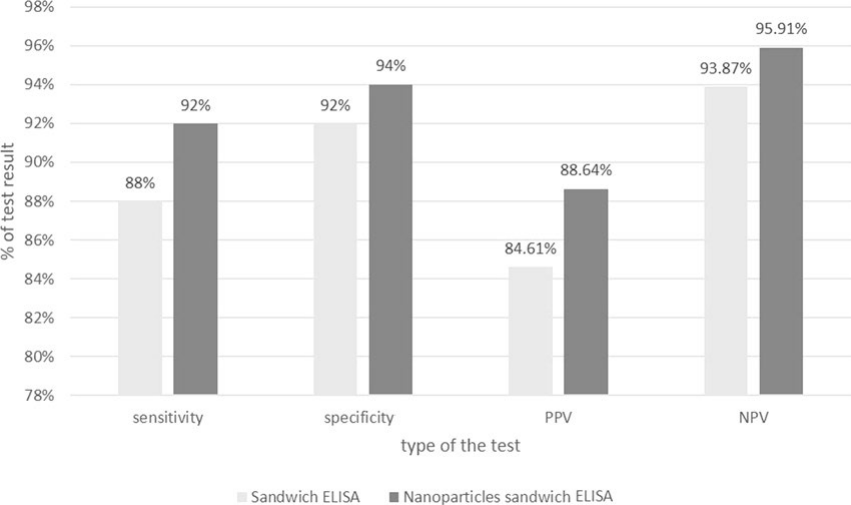
Sensitivity, specificity, positive predictive value (PPV) and negative predictive value (NPV) of both Sandwich ELISA and Nano-Sandwich ELISA.

Standardization of both sandwich ELISA and nano-sandwich ELISA for PGA detection in stool samples showed that sensitivity & specificity of sandwich ELISA were 88% & 92% respectively while by nano-sandwich ELISA they were 92% & 94% respectively. Also, it showed that PPV & NPV of sandwich ELISA were 84.61% & 93.87% respectively while by nano-sandwich ELISA they were 88.64% & 95.91% respectively (Fig. 4).

## Discussion

This study is designed to highlight the importance of using Mini-Parasep SF concentration in antigen detection by conjugated antibodies on AuNPs in sandwich ELISA immunoassay for *G. lamblia* diagnosis. For the last thirty years, fecal concentration tubes were usually used, over this period of time various modifications have been made to the system improving the yield of eggs, cysts, and trophozoites of different parasites of variable sizes and shapes^27^. Closed fecal concentration tubes give an effective satisfactory diagnosis of different intestinal parasites^28^. Concentration methods are modified by using a small size sieve (425 μm) in the Parasep O&P filter fecal concentrator tubes which trap rejected particles and debris in stool samples, preventing their extrusion into the sedimentation cone during centrifugation. Thus improving the clarity results in an increased accuracy of diagnosis during microscopy^29^. In other concentrator tubes, the size of the filter pores may vary between 600 μm and 2000 μm thereby resulting in the passage of debris containing small parasitic forms such as *Entamoeba* and *G. lamblia* cysts which are lost from diagnostic films^13^.

In the current work, we used Mini-Parasep tubes without solvent for health and safety process, as well as eliminating cross-contamination as they are disposable while in another study Alcorfix fixative which was relatively new to North American market was used in processing of specimens added by the Parasep tubes which were attractive tool in parasitology laboratories although no universal comparison was made among methods commonly used in this broad geographic region. To obtain better ova recovery results, a comparison was made between ethyl-acetate precipitation technique with Parasep solvent-free tubes and the standard ethyl-acetate technique in a European study^30^.

Also in another study, the evaluation of the single vial in laboratory methodology of United States laboratories, the Parasep tube was compared to the current standard techniques to improve the performance characteristics of diagnostic techniques using these tubes and fixatives to reach the level of or exceed the current gold standard. The core processes of stool concentration are mostly superimposable or based on conserved methods in spite of using different alternatives of O&P testing by many laboratories in North America^31^.

Looking at the previously reported study^13^, we found that no coccidian oocysts or microsporidian spores could be identified by Mini-Parasep due to their small size and mass, whereby they entrapped in fecal plug and fail to sediment properly so they recommended the use of surfactants such as Triton X-100 thus helping in reducing the surface tension in mucus fecal plug enhancing filtration of parasitic stages that may otherwise remain entrapped in the specimen. The authors mentioned that the use of surfactants was not necessary in Parasep concentration tube as they have already demonstrated a reliable increase in the number of detected parasites and positive tests. In our opinion, a separate trial is needed to establish the role of surfactants. Also this highlighted the valuable use of Mini-Parasep in antigen isolation and detection of parasitic infections instead of direct diagnosis.

In this work we found that Parasep had many advantages especially for developing countries being an alternative for direct microscopy and conventional concentration techniques by providing a hazard-free, reliable and safe method with satisfactory efficacy at improving the parasitic yield in fecal specimens in addition to decreased processing time required which may increase the processing capabilities and output findings of various parasitology tests. This was in agreement with Couturier *et al*.^31^, who achieved their targetable goals for improvement by adding new modifications in the processing of specimens and preparation of concentrates for microscopic examination of specimens. Also increased demand of laboratories for O&P testing emerges two factors that are needed to improve the field of diagnostic methodology, the first is significant challenges in meeting clinically reasonable turnaround times and the second is increased demand of the most experienced parasitologists who are becoming less numerous, therefor Parasep tubes appeared as a distinctive alternative for parasitology laboratories.

In our current work, GA was isolated, purified and analyzed from stool samples thus the process of preparation and purification of GA was followed by preparation of pAb against PGA from rabbit sera. Mini-Parasep (Fecal Parasite Concentrator) was used in the purification of GA from stool samples. Some studies used Bio-Rad Protein Assay in measurement of the crude GA in stool samples being estimated to be 8 mg/ml while the PGA showed four density bands at 47.5, 17.0, 14.0 and 12.5 kDa by using of 12.5% Sodium Dodecyl Sulfate-Polyacrylamide Gel Electrophoresis (SDS-PAGE) technique under reducing condition, as well as indirect ELISA was used for detection of the antigenicity of the crude antigen^13,31^.

A combination of the PGA with complete and incomplete Freund’s adjuvant was made to immunize rabbits and prepare rabbit anti- *G. lamblia* IgG pAb^32^. Many purposes were detected form usage of adjuvants in animal’s immunization protocols including enhancement of the immune response to the antigen, furthermore introducing a depot for the immunogens at the injection site allowing for slow, prolonged release of the immunogens in the animal, this agreed a previously reported work^33^.

Determination of the reactivity of purified IgG pAb (ppAb) against PGA and other parasitic antigens (*Entamoeba histolytica* and *Enterobius vermicularis*) in the present study was made by indirect ELISA thus the primary capture of purified pAb was done to coat ELISA plate and the secondary capture of pAb was done by Horse-Raddish Peroxidase enzyme (HRP) conjugation on ELISA plate. This was in line with Majidi *et al*.^15^, who used sandwich ELISA captured with purified HRP-labelled IgG pAb for determination of reactivity of ppAb against 65 kDa *G. lamblia*-specific stool antigen (GSA) and other parasitic antigens (*Entamoeba histolytica* and *Blastocystis*) and for recognition of 65 kDa GSA as a coproantigen in patients’ stool specimens. They also recognized an optimum dilution of 1/100 for ppAb as a coating layer whereas dilution of 1/50 for ppAb as a peroxidase-conjugated layer.

In our study, the purification steps were satisfactory and ppAb assessment was made by 12.5% SDS-PAGE thus we used two purification methods including ammonium sulfate precipitation and 7% caprylic acid according to a previously reported study^34^. These purification procedures gave a protein product content of pAb as 3.5 mg/ml IgG which decreased from the starting content of 11 mg/ml before purification, on using these procedures with any biological fluid, the immunoglobulin product contents of purification were agreeing to somewhat with previously reported studies^35,36^ while Stec *et al*.^37^, concluded that different protein recoveries were obtained, being 75% of the protein recovered by purification of pAb of two steps procedure (ammonium sulphate precipitation and Protein G Sepharose Chromatography) and being 50% protein product was estimated after the Mono Q column followed by Superdex 200.

Other studies revealed good sensitivity of ELISA as diagnostic test in detection of *G. lamblia* infection^38^. These raised the ceiling of *Giardia* diagnosis to show positive fecal samples even if live parasite is absent^39^. In the same line Jahan *et al*.^40^, compared direct wet mount microscopic diagnosis which revealed only 260 positive cases of giardiasis in relation to 380 cases which were diagnosed positive for *G. lamblia* infection by ELISA test out of 1680 cases.

In the present work, sandwich ELISA revealed a sensitivity level reached to 88% and specificity to 92% in the time that it detected 68 positive cases of giardiasis out of 81 cases, however PPV and NPV were 84.61% and 93.87% respectively lower than the results of Kamel *et al*.^25^, who revealed in his work a sensitivity level was 95.12% and a specificity was 92.85% while PPV and NPV were 92.85% and 95.12% respectively. While these results were in agreement with Al Saeed and Issa^41^ who used ELISA in the detection of *G. lamblia* antigen in stool specimens. Another study had detected higher specificity of sandwich ELISA assay for detection of GA being 93.83%^42^.

Our study aimed at conjugating anti- *G. lamblia* pAb to paramagnetic nanoparticles for PGA detection with a high sensitivity and specificity capabilities and we found achievement of our goal by representing increase in sensitivity to 92% compared to 88% by traditional sandwich ELISA while specificity increased to 94% compared to 92% by traditional sandwich ELISA. Those results were reflected also on the PPV and NPV being increased from 84.61% to 88.64% and from 93.87% to 95.91% respectively. These results disagreed those of Al Saeed and Issa^41^ who detected sensitivity and specificity of giardiasis diagnosis by ELISA as 76.4% and 100% respectively.

68 out of 81 *G. lamblia* infected patients showed positive results by sandwich ELISA while 74 out 81 by nano-sandwich ELISA. All the 20 healthy control showed negative results and the sensitivity in healthy control was 100% while 16 out of 20 of other parasites (group II) were found to be below cut-off value by sandwich ELISA and 19 out of 20 were below cut-off value by nano-sandwich ELISA, giving an overall specificity 92% and 94% respectively. Similar findings were obtained by a previously reported work^43^.

## Conclusion

Advantages of this method compared with existed commercial detection scheme that short assay time and raised accuracy of antigen detection providing concentrated sample without dangers of solvent usage as well as raising the sensitivity and specificity of ELISA through binding AuNPs. This method overcame the low positivity of single samples which was forcing the technician to take 3 consecutive samples. As well as the narrow size distribution of AuNPs increases the chance of antigen detection in concentrated samples thus reducing the lower detection limits. Mini-Parasep concentrator also helps in purification for giardia antigen.
